# Ghrelin’s role in sleep and sleep deprivation: a narrative review

**DOI:** 10.3389/fpsyt.2026.1744781

**Published:** 2026-01-30

**Authors:** Marta Ditmer, Aleksandra Wojtera, Agata Gabryelska, Szymon Turkiewicz, Piotr Białasiewicz, Dominik Strzelecki, Marcin Sochal

**Affiliations:** 1 Department of Sleep Medicine and Metabolic Disorders, Medical University of Lodz, Lodz, Poland; 2 Department of Affective and Psychotic Disorders, Medical University of Lodz, Lodz, Poland

**Keywords:** circadian rhythm, ghrelin, sleep, sleep deprivation, sleep disorders

## Abstract

Deprivation of sleep (DS) is widespread in modern societies and is associated with cardiometabolic, cognitive, and psychiatric disturbances. Acute DS has also been reported to produce short-lived improvements in mood in some individuals with depression, suggesting the involvement of specific biological mediators. Ghrelin, a stomach-derived peptide with central actions in hypothalamic and limbic circuits, has emerged as a candidate linking DS with alterations in sleep, circadian regulation, mood, and cognition. Both acylated and unacylated isoforms exhibit distinct biological activities, and accumulating evidence points to roles in sleep architecture, stress responsivity, and neuroplasticity, as well as in disorders such as insomnia, obstructive sleep apnea, and narcolepsy. Experimental studies indicate that DS frequently coincides with changes in circulating ghrelin, although findings remain heterogeneous and influenced by methodological and contextual factors. Overall, ghrelin may contribute to the pathways through which DS influences emotional regulation and cognitive functioning. A more detailed understanding of its isoform-specific, sex-dependent, and circadian-stage effects could help guide future research and support the development of therapeutic approaches that complement existing strategies for mood and sleep disorders.

## 1 Introduction

Sleep is a fundamental biological process essential for cognitive functioning, emotional regulation, and overall health (1). However, in modern society, insufficient sleep has become increasingly widespread, with over one-third of adults habitually obtaining a sleep duration that deviates from the recommended range by public health authorities for maintaining overall health and well-being (2). This widespread sleep deficiency is even more prominent in specific professions such as healthcare workers, military personnel and shift workers, where chronic sleep deprivation (DS) is often an inherent part of the job (3).

DS is a condition characterized by a forced reduction in the duration of sleep. DS can be caused by external factors, such as work, stress, or environmental disturbances, as well as internal factors, including pain or underlying medical conditions (4, 5). A clear distinction must be made between DS and insomnia, which is a clinical sleep disorder defined by persistent difficulties with sleep initiation and/or maintenance despite favorable sleep conditions (3, 5, 6).

A large body of evidence links DS to adverse health outcomes, including cardiovascular, neurological, endocrine, and immune disorders, as well as increased risk of accidents and impaired cognitive functioning (3, 6, 7). However, paradoxically, acute DS can transiently improve mood, particularly in individuals with depressive disorder (8, 9). According to recent meta-analyses, a positive emotional response to DS is estimated at 50-80% (10). The cognitive consequences of DS may be modulated by an individual’s emotional response to sleep loss. Specifically, individuals exhibiting stable or improved mood following DS tend to perform better on cognitive assessments compared to those whose depressive symptoms aggravated (5). This paradox underscores the importance of identifying the biological mechanisms through which sleep loss influences mood and cognition.

Several candidate mechanisms have been proposed, including alterations in monoaminergic transmission, neurotrophin signaling, inflammatory activity and circadian regulation (11–14). Ghrelin has also emerged as a plausible biochemical mediator of the relationship between sleep loss and mood (15). This neuropeptide, alongside several other hormones regulating hunger and satiety, only recently became a subject of interest in psychiatry largely due to promising findings from studies on the orexin pathway. Orexin receptor antagonists have emerged as key molecules in the psychopharmacology of not only sleep disorders, such as insomnia, but also depression, opening a new avenue for research (16, 17). Even though ghrelin does not directly share a signalling pathway with orexin, they remain tightly connected in regard to their function and anatomical distribution of receptors (18). Given these clinical advances, the functional and anatomical links between ghrelin and orexin raise the possibility that ghrelin-related mechanisms might also be targeted for psychiatric applications, in particular for novel treatments or adjunctive strategies in mood and sleep disorders.

Indeed, studies up to date revealed that ghrelin, besides its influence on metabolism, hunger and satiety, has a complex and multifaceted effect on mental health, including mood regulation and cognitive functions (19–21). Interestingly, ghrelin shows potential as a therapeutic target in depression, where its neuroprotective and mood-modulating properties could be harnessed to improve mental health outcomes (19, 22). Dysregulation of the ghrelin system has been reported in patients with major depressive disorder, bipolar disorder, and eating disorders, suggesting that ghrelin signaling may reflect shared neurobiological pathways linking affective symptoms, energy homeostasis, and sleep disturbances (23). Moreover, experimental manipulation of ghrelin levels has been shown to influence neuroplasticity, stress coping, and hippocampal-dependent memory, all of which are core domains affected in psychiatric illness (24). This neuropeptide was also able to affect sleep architecture, improving its overall quality and efficacy as well as interact with circadian rhythm, which further emphasizes its role in this aspect of functioning (25, 26). Furthermore, ghrelin has been implicated in sleep disorders such as insomnia, obstructive sleep apnea (OSA) and narcolepsy (27–29). DS alters ghrelin signaling, often amplifying its effects on hunger and energy balance, which may further disrupt sleep quality and circadian regulation (30).

This narrative review aims to assess the role of ghrelin in sleep regulation and its involvement in the consequences of DS, with particular emphasis on its potential function as a mediator between mood and sleep loss.

## 2 Ghrelin properties

Ghrelin was first isolated in 1999 by Kojima et al. from rat stomach tissue (31). This 28-amino acid peptide is primarily synthesized by X/A-like cells in the gastric fundus, but it is also expressed in the intestine, pancreas, kidneys, and hypothalamus (32, 33). In humans, the ghrelin gene (GHRL) is located on chromosome 3p25–26 and consists of six exons (34). Biosynthesis begins with transcription and splicing of the GHRL gene, producing a 117-amino acid precursor known as preproghrelin, which contains a signal peptide, the ghrelin sequence, and the C-ghrelin domain (34). After removal of the signal peptide in the endoplasmic reticulum, proghrelin is generated and further processed into unacylated ghrelin (UAG) and C-ghrelin; the latter can be converted into obestatin (34). A critical post-translational step is the acylation of UAG by ghrelin O-acyltransferase (GOAT), which attaches an octanoic acid to serine-3, thereby producing acylated ghrelin (AG) (34). This modification, reversible through the action of circulating esterases, is essential for full biological activity (34, 35). While UAG was once considered inactive, accumulating evidence suggests it contributes to physiological regulation of glucose metabolism, vascular tone and blood pressure, though its receptor remains unknown (36, 37).

AG exerts effects in both peripheral tissues and the central nervous system. Its central actions are facilitated by the ability to cross the blood-brain barrier (BBB) via a saturable, bidirectional, and regulated transport system (32, 38). Complementary evidence also supports active transfer across the blood-cerebrospinal fluid (CSF) barrier, together indicating the presence of multiple entry pathways into the brain (39). Ghrelin’s principal receptor, the growth hormone secretagogue receptor type 1a (GHSR1a), is widely expressed in peripheral tissues and distributed throughout the brain (31). An alternative splice variant, GHSR1b, lacks signaling capacity but may regulate GHSR1a trafficking or availability, or act as a dominant-negative regulator (40–43). GHSR1a is a G-protein-coupled receptor primarily coupled to the Gαq/11 protein complex, which upon activation initiates several intracellular signaling cascades (35, 44). These include MAPK and PI3K/Akt pathways, which could mediate ghrelin’s cardiovascular effects such as endothelial proliferation, survival, and angiogenesis (44). Phospholipase C activation promotes intracellular Ca^2+^ release and CaMK-mediated phosphorylation of AMP-activated protein kinase (AMPK) which links ghrelin signaling to energy regulation as well as cardio-, hepato-, and neuroprotective effects (35, 44). Notably, GHSR1a also displays high constitutive activity independent of ligand binding, which may contribute to regulation of basal growth hormone secretion and body weight (45).

Consistent with its central accessibility, in the hypothalamus, GHSR1a signaling in Neuropeptide Y (NPY)/Agouti-related peptide (AgRP) neurons is required for ghrelin’s orexigenic action (35). Mice with disrupted Gαq/11 signaling in these neurons show attenuated feeding responses to peripheral ghrelin, while deletion of Gα12/13 does not impair this effect (35). Beyond feeding, ghrelin plays a role in emotional regulation and stress adaptation. For example, restraint stress in mice was shown to increase GHSR1a expression in αCaMKII+ neurons of the ventromedial hypothalamus, which promoted anxious behavior (46). However, evidence is mixed: in another study GHSR1a-deficient mice did not show anxiety-like behavior, whereas loss of functional ghrelin signaling has been linked to depressive phenotypes and impaired stress adaptation, suggesting a complex role in emotional resilience (47).

Beyond ghrelin, the GHSR1a is also regulated by liver-expressed antimicrobial peptide 2 (LEAP2), which counteracts ghrelin signaling by acting as both a competitive antagonist and an inverse agonist of the receptor’s constitutive activity (48). LEAP2 is primarily produced in peripheral tissues, particularly the liver and small intestine, but its expression has also been reported in several brain regions (49). Experimental evidence indicates that peripheral LEAP2 reduces food intake, likely through modulation of hypothalamic appetite-regulating circuits (49). Conversely, central LEAP2 signaling may exert effects on reward-related pathways that oppose those of ghrelin, attenuating food-associated reward processing and implicating LEAP2 in the regulation of eating behavior and potentially in eating disorders (49, 50).

In addition to its actions through GHSR1a, ghrelin modulates other molecular networks. Its appetite-stimulating effects involve regulation of sirtuin 1 (SIRT1) and p53 which enhance the expression of orexigenic neuropeptides such as AgRP and NPY in the hypothalamic arcuate nucleus (ARC) (51). Ghrelin has also been shown to influence the mammalian target of rapamycin (mTOR) pathway, a central regulator of metabolism, cell growth, and survival, thereby linking it to conditions including obesity, diabetes, depression, and cancer (44). Collectively, ghrelin emerges as a multifunctional peptide hormone at the crossroads of metabolic, cardiovascular, emotional, and sleep-related processes, acting through an intricate network of endocrine, paracrine and central signaling pathways.

In most experimental and clinical studies, total ghrelin is measured in plasma, serum, or CSF (52, 53), which does not allow differentiation between the acylated and unacylated forms, potentially obscuring their distinct biological effects. Accurate quantification of AG and UAG requires strict pre-analytical control, including the addition of esterase and protease inhibitors to prevent rapid de-acylation, thereby complicating study protocols (54). Although ghrelin can cross the BBB, peripheral concentrations may only partially reflect central levels. This is because BBB transport kinetics, isoform specificity, and individual metabolic factors influence ghrelin’s passage, and, additionally, ghrelin is synthesized within the brain (38, 55). Consequently, peripheral concentrations - whether measured as total ghrelin or separated into AG and UAG - provide only an imperfect proxy for central exposure.

## 3 Ghrelin, sleep and selected sleep disorders

### 3.1 Ghrelin and sleep architecture

Sleep is divided into two main types: non-rapid eye movement (NREM) and rapid eye movement (REM) sleep. NREM consists of three stages, with stage 3 referred to as slow-wave sleep (SWS), the deepest sleep phase (4). SWS is critical for memory consolidation and physical recovery (56). REM sleep, characterized by vivid dreaming and muscle atonia, plays a key role in emotional regulation and cognitive functions (57). Across a typical night, NREM and REM alternate in cycles of roughly 90 minutes, with REM episodes lengthening in the later part of the night (4).

Increasing attention has been directed toward hormonal modulators of sleep architecture, including ghrelin. Ghrelin plays a significant role in regulating sleep, exerting modulatory effects in several brain regions including the hypothalamus, hippocampus, olfactory bulb and occipital cortex (52). Experimental work suggests that reduced central ghrelin levels in smokers may contribute to insufficient sleep, implying that maintaining an appropriate plasma-CSF ghrelin balance is important for healthy sleep regulation (52).

Evidence from human studies indicates that ghrelin functions as an endogenous sleep-promoting factor in males (58). Since aging is naturally accompanied by alterations in sleep structure, duration, and quality—largely driven by circadian rhythm changes and ghrelin’s interactions with clock genes—one might expect advancing age to modulate its effects on sleep (59, 60). Nonetheless, available data suggest that ghrelin’s impact on sleep in men remains relatively stable across the lifespan. Weikel et al. demonstrated that ghrelin administration in healthy young men promoted SWS while reducing REM sleep during the second third of the night (25). Supporting these findings, Kluge et al. reported that ghrelin administration in young males resulted in an increase in NREM sleep, especially through a greater amount of stage 2 sleep during the latter part of the night, along with a reduction in REM sleep (61). Studies in elderly men have shown comparable results: ghrelin administration increased stage 2 sleep, SWS, and total NREM sleep, while reducing stage 1 and REM sleep (62).

In contrast, ghrelin had no measurable effect on sleep architecture in either young or elderly women, indicating that ghrelin’s influence on sleep appears to be sex-dependent rather than age-dependent (62, 63).

Sex-related differences in the effects of ghrelin on sleep architecture between young men and premenopausal women may be explained by several factors, one of which is the complex and still poorly understood interaction between ghrelin and female sex hormones. Plasma ghrelin levels are generally higher in women than in men, although in healthy women circulating concentrations of total ghrelin, AG, and UAG do not vary significantly across the menstrual cycle (64–67). These observations raise the possibility that ghrelin receptor sensitivity, particularly within signaling pathways directly or indirectly implicated in sleep regulation, differs between sexes and may be further modulated by menstrual cycle-dependent hormonal fluctuations. Animal studies have demonstrated that ghrelin receptor responsiveness varies across the estrous cycle, and small-scale human investigations suggest a comparable pattern across the menstrual cycle, particularly in relation to prolactin (PRL) (67–69). Ghrelin stimulates PRL secretion via its primary receptor, GHSR1a (68). PRL, in turn, exhibits a bidirectional relationship with sleep (70). Its secretion follows a distinct circadian rhythm, characterized by elevated nocturnal levels and reduced daytime concentrations (70). In rodent models, hyperprolactinemia has been closely associated with enhanced REM sleep, and PRL release correlates strongly with delta power- an electrophysiological index of sleep depth and homeostatic sleep pressure (70). In human experimental studies, Messini et al. reported that administration of ghrelin at a dose of 0.30 μg/kg body weight significantly increased circulating PRL concentrations only during the late follicular phase, whereas growth hormone (GH) - another peptide regulated by ghrelin through GHSR1a - remained unchanged (69). In contrast, their earlier study employing a higher ghrelin dose (1 μg/kg) did not reveal significant alterations in GH or PRL secretion across menstrual phases (69). These findings support the hypothesis that menstrual cycle-related hormonal variations can influence ghrelin receptor sensitivity and thereby modulate certain downstream pathways involved in sleep regulation. Furthermore, the physiological variation in PRL secretion across different phases of the reproductive cycle adds an additional layer of complexity to the interpretation of these interactions (70).

The absence of significant changes in sleep architecture observed in young women compared with men following exogenous ghrelin administration may therefore reflect underlying sex-specific regulatory mechanisms. One plausible explanation is that the chronically higher endogenous ghrelin exposure in females leads to a compensatory reduction in ghrelin receptor sensitivity or expression, attenuating the physiological response to exogenous stimulation. In addition, fluctuations in gonadal hormones across the menstrual cycle may dynamically influence ghrelin receptor function or its downstream signaling pathways relevant to sleep regulation. Future research in women should therefore account for menstrual cycle phase and explore a range of ghrelin doses, as a mere increase in the administered dose may not necessarily enhance efficacy.

With regard to differences between postmenopausal women and elderly men, the underlying mechanisms remain unclear. Plasma concentrations of AG do not differ significantly between the sexes in older age (71). After menopause, circulating levels of female sex hormones diverge only slightly from those in men, making them unlikely contributors to sex-related differences in ghrelin’s effects on sleep (72).

Sexually dimorphic effects of ghrelin but also of GHRH and possibly other hormones on sleep may contribute to the consistent finding that sleep differs between sexes across lifespan (73). Considering that AG and UAG exert distinct physiological effects within the central nervous system, including opposing actions on neurogenesis and memory function (74), it is crucial to investigate their specific contributions to sleep regulation. Since studies on ghrelin’s role in sleep architecture have so far been conducted on relatively small samples, up to 20 participants, future research involving larger study populations is needed to obtain more reliable and generalizable results.

### 3.2 Ghrelin and circadian rhythm

The circadian rhythm is an internal, near-24-hour cycle regulating various physiological processes, including sleep, metabolism, and behavior. Its central clock, known as the suprachiasmatic nucleus (SCN), is located in the hypothalamus and is programmed to synchronize bodily functions with the external day-night cycle (75). Peripheral clocks are located in the tissues and control circadian processes on a cellular level and are capable of maintaining autonomous rhythmicity. These peripheral oscillators are coordinated by the central clock but also respond to local cues (75).

The molecular basis of circadian regulation is controlled by a transcriptional-translational feedback loop involving several core clock genes. Circadian Locomotor Output Cycles Kaput (CLOCK) and Brain and Muscle ARNT-Like Protein-1 (BMAL1) initiate the expression of Period Circadian Regulator 1 (PER1) and Cryptochrome Circadian Regulator 1 (CRY1) genes, whose protein products inhibit CLOCK and BMAL1 activity, forming a self-regulating loop (12). Additional regulators, such as Nuclear Receptor Subfamily 1 Group D Member 1 (NR1D1), which suppresses the expression of BMAL1 and CLOCK, and Neuronal PAS Domain Protein 2 (NPAS2), which can substitute for CLOCK by forming a heterodimer with BMAL1, further modulate these pathways and contribute to the complex regulation of circadian timing (12).

Anatomical studies reveal that ghrelin signaling is closely linked to brain regions involved in circadian regulation. The study conducted by Zigman et al. on rodents demonstrated that GHSR1a is robustly expressed in the SCN (76). Furthermore, ghrelin-immunoreactive neurons are distributed across several hypothalamic nuclei involved in circadian output, including ARC, paraventricular, dorsomedial and ventromedial nuclei (77). Among these regions, the ARC is of particular importance, as it serves as a critical integrative center for coordinating metabolic cues with circadian regulatory mechanisms by maintaining a close, reciprocal relationship with the SCN (78).

Plasma ghrelin levels exhibit a clear circadian pattern in both rodents and humans; its levels typically rise before meals and fall after food intake, indicating their role in meal anticipation (79, 80). Mice deficient in BMAL1 fail to exhibit the normal circadian pattern of plasma ghrelin, implying that ghrelin release is not solely dependent on external cues like feeding but also governed by intrinsic circadian mechanisms (80). Moreover, ghrelin itself appears to influence the circadian rhythm, it has been shown to modulate the expression of PER1 and Period Circadian Regulator 2 (PER2) in peripheral tissues like the stomach, suggesting a bidirectional interaction (81). In a pig model, Zhang et al. demonstrated that ghrelin could modulate the circadian phase under irregular eating patterns, even when the total daily food intake remains constant (82). Ghrelin was found to enhance mRNA expression of core molecular clock genes, including positive (BMAL1, CLOCK) and negative (PER1, PER2, CRY1, Cryptochrome Circadian Regulator 2) elements (82). These findings highlight ghrelin’s potential role in aligning feeding cues with circadian gene expression, particularly under conditions of irregular eating, and support the idea that ghrelin and the circadian system interact bidirectionally (82).

Experimental evidence indicates that ghrelin is not only a downstream effector of circadian timing but also an active modulator of the central clock itself. *In vitro* application of ghrelin to SCN tissue was able to shift the circadian phase by up to 3 hours (83). When administered during the subjective day, ghrelin also increased the expression of PER2::LUCIFERASE, a fusion protein which might serve as an indicator of circadian dynamics (83, 84).

Shift work profoundly disrupts the circadian system by forcing individuals to remain active, eat, and sleep at times misaligned with their endogenous biological rhythms (85). This misalignment leads to desynchronization between the central clock and peripheral clocks in metabolic tissues (85). Exposure to light at night, a hallmark of night shift work, delays circadian phase, which shortens sleep duration and impairs sleep efficiency (85). Such internal desynchrony may undermine the coordinated timing of physiological processes that maintain metabolic stability. Simulated night shift protocols in humans’ studies showed that circadian misalignment elevates the 24-hour and postprandial AG levels, with this effect persisting over time (86, 87). Such sustained increases may reflect a failure to adapt to circadian disruption. This observation suggests that ghrelin could contribute to the metabolic and sleep disturbances commonly observed in shift workers and other populations exposed to circadian misalignment.

In summary, ghrelin and the circadian system are closely linked through a bidirectional relationship that extends beyond ghrelin’s established role in metabolic regulation. Ghrelin not only exhibits circadian rhythmicity but also actively participates in modulating central and peripheral clocks, thereby acting as an internal regulator of circadian timing. This interplay suggests that ghrelin may play a critical role in maintaining synchrony between feeding behavior and biological rhythms, particularly under conditions of circadian disruption, such as shift work or irregular meal timing. Future research should investigate the relationship between endogenous or exogenously administered ghrelin levels and the expression of core clock genes in humans under both physiological and pathological conditions, as evidence in this area remains limited.

### 3.3 Ghrelin and insomnia

Insomnia is the most prevalent sleep disorder among adults, affecting approximately 6-10% of the population chronically and up to 36% experiencing occasional symptoms (88, 89). Chronic insomnia disorder is defined as difficulty initiating or maintaining sleep occurring at least three nights per week for a duration of three months or longer (89). Insomnia has been consistently linked to poorer physical and mental health outcomes, including impaired quality of life, memory problems, and decreased concentration. It is also accompanied by daytime symptoms such as fatigue, irritability, and cognitive impairment (89).

Recent studies suggest that ghrelin may play a role in the pathophysiology of insomnia. Motivala et al. found that patients with chronic insomnia had significantly lower nocturnal ghrelin levels, approximately 30% lower, compared to age- and weight-matched healthy control subjects (27). Additionally, chronic insomnia has been associated prospectively with weight gain and cross-sectionally with obesity, conditions that are also influenced by alterations in ghrelin levels (27). For a summary, see **Table 1**.

**Table 1:** Summary of selected studies on the subject of ghrelin in sleep disorders.

Sleep disorder	Author	Sample	Biomaterial; Time of collection	Main findings
Insomnia	Motivala et al. (27)	14 men with primary insomnia and 24 matched controls	Plasma; 11PM, 2AM, 6AM	In individuals with chronic insomnia, nocturnal ghrelin levels were reduced by about 30% relative to controls matched for body weight.
OSA	Harsch et al. (28)	30 patients with OSA and 30 matched controls	Plasma; 7:15AM	Plasma ghrelin concentrations were significantly higher in OSA patients compared to BMI-matched controls.
	Yang et al. (90)	25 patients with OSA, 25 patients with OSA and CHD, 25 patients with CHD, and 25 matched controls; 23 men and 2 women per group	Plasma; after overnight fasting (exact time not specified)	Fasting plasma ghrelin levels were higher in OSA patients compared to controls.
	Golshah et al. (91)	meta-analysis including >1000 participants (OSA patients and matched controls)	Serum and plasma; various collection times	Initial analysis showed no significant differences; however, after excluding outlier studies, ghrelin levels were significantly higher in adults with OSA compared to controls.
	Pardak et al. (92)	41 male, 5 female OSA patients and 12 matched controls	Plasma; 5 AM and 7 AM	Plasma ghrelin levels were lower in OSA patients at early morning time points; levels were lower in OSA patients with obesity compared to obese controls and non-obese OSA patients.
	Liu Weiying et al. (93)	95 male OSAS patients, 30 BMI-matched non-OSAS subjects, 30 normal-weight controls	Plasma; 7 AM and 8 AM	Compared with normal-weight controls, morning plasma ghrelin was significantly reduced in participants with OSAS and in BMI-matched non-OSAS participants. Ghrelin concentrations correlated negatively with BMI.
	Zhang et al. (94)	30 male patients with severe OSA and 20 matched controls	Plasma; 6 AM and 7 AM	No significant differences in plasma ghrelin levels were observed between OSA patients and controls.
Narcolepsy	Donjacour et al. (95)	8 male patients with hypocretin-deficient narcolepsy with cataplexy, and 8 matched controls	Plasma; 24-h blood sampling at 60-min intervals	Diurnal ghrelin profiles did not differ significantly between narcolepsy patients and controls, basal ghrelin secretion was preserved.
	Huda et al. (29)	4 male and 4 female patients with chronic, severe narcolepsy-cataplexy, 8 matched controls	Plasma; baseline, 30, 60 (preprandial), 90, 120, 180 and 240 min (postprandial)	Meal-related plasma ghrelin concentrations were comparable between narcolepsy patients and controls.
DS	Schmid et al. (96)	9 men	Plasma; 7:00 AM and 7:30 AM	A single night of DS significantly increased plasma ghrelin concentrations compared to normal sleep.
	Hogenkamp et al. (97)	16 men	Plasma; 7:30 AM	Plasma ghrelin concentrations were higher after one night of DS compared to a night of normal sleep.
	Chapman et al. (98)	14 men	Plasma; 7:30 AM	Morning plasma ghrelin levels were significantly elevated following DS compared to normal sleep.
	Egmond et al. (99)	24 men, 20 women	Plasma; 7:30 AM	DS increased morning plasma ghrelin concentrations in both sexes, with a more pronounced effect in individuals with obesity.
	Dzaja et al. (100)	10 men	Plasma; 24-h blood sampling at hourly intervals	DS disrupts ghrelin rhythmicity, blunting the normal early-night rise and instead slightly elevating levels throughout the night until morning.
	Schüssler et al. (101)	5 men, 3 women	Plasma; every 30 min between 8 PM and 10 PM and every 20 min between 10 PM and 7AM (also night after DS)	Elevated ghrelin concentrations during the recovery night following DS were observed, suggesting a role of ghrelin in the regulation or promotion of restorative sleep.
Sleep Restriction	Spiegel et al. (102)	9 men	Plasma; 20-min intervals from 8:00 AM to 9:00 PM	Elevated plasma ghrelin concentrations were observed after sleep restriction to 4 h sleep/night for 2 nights compared to normal sleep.
	Lin et al. (103)	meta-analysis including 2.250 participants	Serum/plasma; various collection times	Significant increase in ghrelin levels were noted in individuals with total sleep time <5h/day.
	Taheri et al. (104)	721 participants	Serum; unspecified	Restricting sleep to 5 hours per night (vs. 8 hours) was associated with higher circulating ghrelin.
	Bosy-Westphal et al. (105)	14 women	Plasma; between 7:00 AM and 11:00 AM	After 4 consecutive nights of restricted sleep (≤4 h per night), circulating ghrelin concentrations did not change significantly.
	Schmid et al. (106)	15 men	Plasma; 7:40 AM, then hourly between 8:00 AM and 11:00 PM	No significant changes in ghrelin concentrations were observed after sleep restriction to 4 h of sleep per night for 2 consecutive nights.

BMI, body mass index; CHD, coronary heart disease; DS, deprivation of sleep; OSA, obstructive sleep apnea; OSAS, obstructive sleep apnea syndrome.

Intervention studies have explored the modulation of ghrelin through dietary strategies. Keser et al. evaluated the impact of a bedtime snack consisting of either milk or banana, both of which are rich sources of the essential amino acid tryptophan - a metabolic precursor of serotonin and subsequently melatonin. Although exogenous melatonin is not routinely recommended as a treatment for insomnia, endogenous melatonin plays a key role in regulating circadian rhythms and facilitating sleep initiation (107–109). In patients with primary insomnia, the authors reported that serum daytime ghrelin concentrations decreased significantly only in the milk group compared to baseline, and this effect was accompanied by improved sleep quality (108). The differences observed between groups may be influenced by sex distribution: the banana group included six women and one man, while the milk group included five women and two men. Previous studies on sleep architecture have shown significant sex-related differences, although the underlying mechanisms remain unclear (63). Moreover, the study was limited by its small sample size (21 patients in total) and self-reported food intake (108). Nevertheless, these findings may encourage future research to investigate whether specific dietary components can modulate ghrelin levels in ways that promote sleep efficiency.

Currently, several effective pharmacological treatments for insomnia act by antagonizing orexin receptors (16). Clinical trials and meta-analyses have demonstrated that orexin receptor antagonists reduce sleep onset latency and improve sleep maintenance, with favorable safety profiles (16, 110). Both orexin and ghrelin are peptide hormones that regulate energy balance, arousal, and sleep-wake dynamics, suggesting that modulation of ghrelin signaling might also be beneficial in this group of patients. However, knowledge regarding ghrelin’s therapeutic potential in insomnia remains very limited, even though it is well documented to influence sleep in a sex-dependent manner (see section 2.1 Ghrelin and sleep architecture). To date, the only study addressing this subject, conducted by Denny et al., reported that an oral ghrelin receptor inverse agonist, PF-05190457, induced dose-dependent somnolence in healthy volunteers, indicating a possible role in disorders such as insomnia (111). At first glance, this finding appears paradoxical, since chronic insomnia is characterized by reduced ghrelin levels (27), whereas further lowering ghrelin signaling pharmacologically produced sedative effects. This apparent contradiction may reflect the complexity of ghrelin’s central versus peripheral actions, or differences between physiological alterations in endogenous ghrelin levels and the pharmacological targeting of its receptor system (112). It also needs to be noted that evidence regarding the ability of PF-05190457 to penetrate the brain remains inconsistent. Moreover, although the compound has been detected in animal brains following systemic administration, it is still unclear whether it acts as a ligand at central GHSRs. Despite ghrelin’s intriguing similarity to orexin-based mechanisms, the evidence base is currently restricted to a single study, and further research is essential to clarify whether ghrelin modulation can offer a viable complement or alternative to orexin receptor antagonism.

Increasing evidence points to a role of ghrelin dysregulation in pathophysiology of insomnia, with lower nocturnal ghrelin levels associated with altered sleep duration. This decrease may result from impaired circadian regulation, leading to blunted nocturnal ghrelin secretion. The current body of research remains limited, highlighting the need for additional, larger, and well-controlled trials. Future trails should further elucidate the bidirectional relationship between ghrelin and sleep, evaluate targeted dietary and pharmacological interventions, and determine whether modulating ghrelin levels in individuals with insomnia can produce sustained improvements in sleep quality and overall health.

### 3.4 Ghrelin and obstructive sleep apnea

OSA is a common sleep-related breathing disorder, affecting up to 36% of adults in the United States and up to 17% of adults in Europe (113), and characterized by repetitive episodes of upper airway obstruction during sleep. These episodes lead to intermittent hypoxia, a pattern of recurrent reductions in blood oxygen levels followed by reoxygenation (114). This cycle of oxygen desaturation and restoration triggers oxidative stress, systemic inflammation, sympathetic nervous system overactivity, and endothelial dysfunction (114). In addition to sleep fragmentation and excessive daytime sleepiness, OSA is also associated with a wide array of adverse health outcomes, including cardiovascular disease, metabolic disturbances, cognitive impairments, and mood disorders (113, 115). Ghrelin, a hormone involved in hunger regulation, energy balance, and potentially responsive to hypoxia, appears to be altered in individuals with OSA (116). Since OSA is often associated with obesity and energy metabolism, understanding ghrelin dynamics in OSA patients has become a focus of recent research, although study results have been inconsistent. Findings are summarized in **Table 1**.

Several studies have reported elevated circulating ghrelin levels in untreated OSA patients compared to healthy controls. For example, Harsch et al., studied 60 men with obesity and observed significantly higher plasma ghrelin concentrations at 7:15AM in OSA patients compared to BMI-matched individuals without the condition (28). Similarly, Yang et al., in a study of 100 overweight men, reported higher plasma ghrelin levels after overnight fasting in OSA patients (90). A meta-analysis of 15 studies on serum and plasma ghrelin concentrations initially found no significant differences between OSA patients and healthy controls; however, after excluding outlier studies, ghrelin levels were significantly higher in adults with OSA compared to controls (91).

Interestingly, not all research points in the same direction. Some studies, such as those by Pardak et al., including 58 men with obesity or overweight, reported lower plasma ghrelin levels in OSA patients compared to controls, particularly at early morning time points, at 5AM and 7AM (92). Moreover, ghrelin levels in patients with both OSA and obesity were lower than those in obese controls, as well as significantly lower than levels in non-obese OSA patients (92). Liu et al., examined men with obstructive sleep apnea syndrome (OSAS, defined as OSA with clinical symptoms such as excessive daytime sleepiness), comparing overweight OSAS patients (n=95) with age- and BMI-matched non-OSAS adults (n=30) and normal-weight controls (n=30). Morning plasma ghrelin was significantly lower in both the OSAS group and their overweight non-OSAS counterparts than in the normal-weight controls. Ghrelin concentrations were inversely correlated with BMI, indicating that suggesting that excessive weight is the main factor influencing ghrelin levels in OSA (93). Other studies have reported no significant differences in ghrelin levels between OSA patients and healthy controls, including research in 50 participants in which plasma samples were collected at 6 AM and 7 AM (94).

Hypoxia appears to be a key pathophysiological factor linking obstructive sleep apnea (OSA) and ghrelin regulation, although findings across studies remain highly inconsistent. Debevec et al. reported that hypoxia reduces circulating acylated ghrelin concentrations (116), whereas Harsch et al. observed a positive correlation between plasma ghrelin levels and minimal oxygen saturation (28), potentially explaining the lower ghrelin levels reported in some OSA cohorts.

Beyond direct effects on ghrelin secretion, OSA is characterized by chronic exposure to intermittent hypoxia and sleep fragmentation, which promote oxidative stress, systemic inflammation, sympathetic activation, and metabolic dysregulation (90, 114). In line with this, a meta-analysis by Nadeem et al. demonstrated a shift toward a pro-inflammatory cytokine profile in individuals with OSA (117). Given that ghrelin exhibits anti-inflammatory properties by suppressing pro-inflammatory cytokine expression in both human and animal models (90), elevated ghrelin levels observed in some patients with OSA may represent a compensatory response aimed at mitigating inflammation induced by intermittent hypoxia. In summary, the direction of changes in circulating ghrelin levels may depend on the relative contribution of underlying mechanisms, potentially reflecting either a compensatory response to inflammation or a hypoxia-associated reduction in ghrelin secretion.

Regardless of the direction of ghrelin concentration changes, continuous positive airway pressure (CPAP) therapy, the standard treatment for OSA that reduces intermittent hypoxia and sleep fragmentation, has been reported to normalize circulating ghrelin levels. Several studies reported a significant decrease in plasma ghrelin following CPAP treatment, with levels approaching those seen in non-OSA subjects (28, 118, 119). However, there are some inconsistencies in the findings, as a meta-analysis of 15 studies showed no significant differences in serum and plasma ghrelin levels in adults with OSA before compared to after CPAP therapy (91).

Taking all these studies into consideration, the divergent results likely stem from a combination of methodological and population-related factors. Firstly, OSA is strongly associated with obesity, which serves both as a major risk factor for its development and as a consequence of sleep fragmentation and metabolic dysregulation (28, 92). Ghrelin has also been implicated in obesity, as its chronic elevation promotes positive energy balance and weight gain (120), making it difficult to disentangle OSA-specific effects from obesity-driven hormonal changes. Secondly, variations in study design: cross-sectional, case-control, or pre-post intervention; may also contribute to inconsistent findings. Thirdly, differences in assay methods, type of ghrelin measured and severity of hypoxia further complicate comparisons. Lastly, the relatively small sample sizes and heterogeneity across studies contribute to high variability; the meta-analysis noted considerable heterogeneity (I² up to 80%) and called for more rigorous, larger-scale studies (91). Moreover, OSA itself appears to encompass a range of phenotypes that differ in symptoms such as insomnia, daytime sleepiness, and comorbid depression (121). Such diversity complicates the assessment of ghrelin levels, as different phenotypes may exhibit distinct pathophysiological mechanisms, highlighting the importance of accounting for these differences in future research.

These discrepancies between studies underscore the need for standardized research protocols and for analyzing ghrelin in relation to BMI, phenotypes and apnea-hypopnea index (AHI), which reflects the severity of OSA, in order to clarify its exact role in OSA pathophysiology and its potential as a therapeutic target.

### 3.5 Ghrelin and narcolepsy

Narcolepsy is a chronic neurological disorder characterized by excessive daytime sleepiness, abnormal REM sleep, and, in the case of type 1 narcolepsy, cataplexy - a sudden loss of muscle tone triggered by strong emotions (122). The disorder is strongly associated with a deficiency of orexin, a neuropeptide produced in the hypothalamus that regulates arousal, wakefulness, and appetite (122). Beyond sleep-wake instability, narcolepsy is often accompanied by metabolic alterations, including an increased risk of obesity and insulin resistance (122). Moreover, orexin neurons are directly responsive to ghrelin stimulation (95). These findings have led researchers to investigate whether hormones involved in energy balance, particularly ghrelin, may contribute to the pathophysiology of narcolepsy.

Several studies have examined whether circulating ghrelin levels differ between patients with narcolepsy and healthy controls. Donjacour et al., examined 24-hour plasma ghrelin concentrations in eight male narcolepsy patients with orexin deficiency compared to eight matched healthy controls (95). There were no differences in mean ghrelin levels across the day between patients and controls, and the normal circadian rhythm of ghrelin secretion was preserved (95). Furthermore, sodium oxybate treatment, widely used to improve nocturnal sleep in narcolepsy, did not significantly alter ghrelin levels (95). The authors thus concluded that increased BMI in narcolepsy is unlikely to be explained by ghrelin dysregulation (95). Meal-related ghrelin responses were examined by Huda et al., who found no significant differences in fasting plasma ghrelin concentrations between narcolepsy patients and controls (29). Importantly, postprandial suppression of ghrelin after food intake was intact in the study group, indicating that dynamic regulation of ghrelin is preserved despite orexin deficiency (29); see **Table 1** for summary. It appears that while orexin neurons normally respond to ghrelin signaling, the loss of orexin in narcolepsy does not disrupt peripheral ghrelin dynamics. However, there are no studies analyzing ghrelin levels in CSF of patients with narcolepsy, which, considering that biologically active form of ghrelin, AG, might be produced in the central nervous system, substantially limits insight into the interplay between ghrelin and sleep-wake regulation.

Studies conducted to date have not demonstrated significant differences in circulating ghrelin levels between patients with narcolepsy and healthy individuals. This supports the hypothesis that ghrelin concentrations are comparable in both groups and are not substantially influenced by the condition, thus several potential implications can be considered. First, it indicates that the increased prevalence of obesity and metabolic syndrome in narcoleptic patients is unlikely to be caused by abnormal ghrelin dynamics. Instead, weight gain may be more closely linked to reduced energy expenditure due to fragmented sleep, lower physical activity, and impaired orexin-mediated sympathetic tone. Second, although ghrelin normally stimulates orexin neurons, the loss of these neurons in narcolepsy suggests a possible functional disconnection. Thus, even if circulating ghrelin levels are preserved, the absence of orexinergic targets may limit its wake-promoting and metabolic effects, pointing toward a central rather than peripheral alteration in ghrelin signaling. Third, the observation that postprandial ghrelin suppression appears preserved implies that peripheral appetite-regulatory mechanisms remain largely intact. This could hold clinical relevance, as dietary interventions and caloric restriction strategies are likely to remain effective in supporting weight management in narcoleptic patients, despite their increased susceptibility to obesity.

Although peripheral ghrelin secretion appears normal in narcolepsy, the downstream hypothalamic pathways that mediate its effects may be dysfunctional (95, 123). Functional neuroimaging, studies involving CSF analysis, and animal models could help clarify whether ghrelin signaling is blunted centrally in patients with narcolepsy, particularly given that orexin deficiency is consistently measured in the CSF (124). Additionally, given ghrelin’s immunomodulatory and neuroprotective properties, its potential contribution to autoimmune mechanisms hypothesized in narcolepsy onset warrants further exploration.

## 4 Ghrelin and DS

### 4.1 DS effects on ghrelin levels

Ghrelin, best known for its role in appetite regulation, also exerts significant effects on brain function, influencing mood, stress responsivity, and reward processing (21). Sleep deficiency, either in the form of complete sleep elimination as DS or through partial restriction of total sleep duration, alters circulating ghrelin levels. Such changes may not only disrupt energy homeostasis but also contribute to alterations in the severity of depressive symptoms and cognitive functions (19, 23). Exploring these interactions provides critical insights into the neuropsychiatric consequences of different forms of sleep deficiency.

Several studies have demonstrated that DS leads to elevated plasma ghrelin concentrations, particularly in the early morning. Schmid et al. reported that even a single night of DS in 9 normal-weight men resulted in a significant increase in plasma ghrelin concentrations measured at 7 AM and 7:30 AM (96). These results are consistent with findings from Hogenkamp et al., who showed that in 16 normal-weight individuals, plasma ghrelin concentrations were higher after a night of DS compared to a night of sleep, with blood collection also set at 7:30 AM (97). Chapman et al. further confirmed this effect in a study of 14 normal-weight men, reporting increased morning (7:30 AM) ghrelin levels after DS (98). Moreover, research involving 44 young adults (24 men, 20 women) with different body weights confirmed that DS raises morning (7:30 AM) plasma ghrelin regardless of sex, with the increase being more pronounced in individuals with obesity (99). Additionally, Dzaja et al. reported that, whereas during normal sleep, ghrelin follows a bell-shaped pattern, rising in the early part of the night and declining toward morning, nocturnal wakefulness blunts this rise, instead increasing ghrelin levels slightly until the morning (100).

However, the evidence on the effects of restricted sleep duration on ghrelin levels remains inconclusive. Spiegel et al. observed elevated ghrelin concentrations after participants (9 healthy men) were limited to four hours of sleep per night over two consecutive days (the blood samples were collected at 20-minute intervals from 8 AM to 9 PM) (102). Similarly, in a meta-analysis including 2.250 participants, Lin et al. reported a significant increase in ghrelin levels among individuals with a total sleep time of less than five hours per day (103). A large-scale study involving 721 participants further confirmed that sleeping only five hours was associated with higher serum ghrelin levels, which the authors suggested may contribute to the increased BMI often observed in chronically sleep-deprived individuals (104). However, not all studies are consistent. In a study involving 14 healthy women, Bosy-Westphal et al. reported no significant changes in circulating ghrelin concentrations measured between 07:00 and 11:00 following four consecutive nights of sleep restriction (limited to a maximum of 4 hours per night) (105). Likewise, another study on 15 healthy men reported that limiting sleep to four hours per night for two nights did not alter circulating plasma ghrelin concentrations (blood samples were collected at 7:40AM and thereafter at 1-h intervals between 8 AM and 11 PM), leading the authors to propose a dose-dependent relationship between sleep reduction and ghrelin levels (106). The apparent discrepancies across studies may be explained by the fact that partial sleep restriction and acute DS differ in several biological aspects, including their impact on circadian regulation. Prolonged sleep restriction, unlike acute DS, can produce distinct changes in the expression of circadian clock genes such as Period 1 (PER1). Insomnia has been linked to reduced PER1 expression, while acute DS has been shown to increase it (12, 125). Similar opposing effects may also be seen in other circadian rhythm genes, depending on the form of sleep loss. These molecular changes could, in turn, affect ghrelin production (12, 125).

The effects of DS on ghrelin may also extend into the recovery phase. In Schüssler et al., the ghrelin peak occurred significantly earlier during the recovery night after DS than at baseline after a night of normal sleep, suggesting a role for ghrelin in the regulation or promotion of restorative sleep (101).

To sum up, the evidence regarding the impact of DS or insufficient sleep on circulating ghrelin levels is compelling but heterogeneous, with outcomes varying according to the specific intervention. Majority of studies support the notion that DS as well as even short-term partial restrictions of sleep duration increase ghrelin concentrations, particularly in the morning. Such changes may reflect a compensatory response to increased total daily energy expenditure. In a study by Markwald et al., insufficient sleep increased participants’ total daily energy expenditure by approximately 5%; however, their energy intake exceeded the amount required to maintain balance (126). This finding suggests a disruption of the leptin-ghrelin regulatory loop, which under normal conditions functions to control appetite and maintain body weight.

Nevertheless, several investigations on the impact of sleep restriction on ghrelin report null or even suppressive effects. These discrepancies may arise from methodological differences, including duration of sleep restriction, sample size, sex distribution, participant BMI, study design, and the timing of blood collection. Ghrelin exhibits a strong circadian rhythm, and subtle variations in sampling windows can lead to divergent findings. Moreover, individual metabolic states and energy balance may further modulate ghrelin responses, highlighting the complexity of these interactions.

### 4.2 Ghrelin’s potential role as a mediator between DS, mood, and cognitive functions

DS has long been recognized for its transient antidepressant effects, with some individuals experiencing rapid mood improvement after even a single night of lost sleep (10). The underlying biological mechanisms remain unclear, but ghrelin, a pleiotropic hormone with both metabolic and neuropsychiatric actions, has emerged as a promising candidate mediator.

Evidence suggests that ghrelin may be involved in the pathophysiology of mood disorders. Individuals with depression have been reported to exhibit elevated circulating ghrelin relative to healthy controls, studies also described positive associations between ghrelin concentrations and depression severity (23, 24). These observations may potentially indicate that increased ghrelin could represent a compensatory, stress-adaptive response to the neurobiological burden of depression. Experimental data further suggest that ghrelin’s antidepressant-like effects involve promotion of hippocampal neurogenesis, paralleling actions reported for conventional antidepressants (127). Consistent with enhanced plasticity, ghrelin increases dendritic spine density and facilitates long-term potentiation in the hippocampus, processes that support learning and memory (128). Some studies also report that antidepressant treatment is associated with lower ghrelin levels (23). Nevertheless, the literature is heterogeneous, with findings ranging from no difference to decreased circulating ghrelin in depressed samples (24). Methodological variability- including specimen type, pre-analytical handling (e.g., storage temperature), and assay characteristics- likely contributes to these discrepancies (23). Additionally, depression is etiologically and phenotypically heterogeneous, with variation in symptomatology, comorbidities (including eating disorders), and other modifiers, which may further shape ghrelin dynamics.

Cognitive impairments are an important dimension of mood disorders, affecting memory, executive function, attention, and processing speed. Up to 85-94% of individuals experience some form of cognitive dysfunction during an acute depressive episode, and 39-44% continue to show deficits even after symptomatic recovery (129). Ghrelin’s effects on cognition have been documented in both animal and human studies, though findings remain inconsistent. In rodents, ghrelin enhances hippocampal function, whereas in humans, Kunath et al. found no cognitive benefits following intravenous ghrelin administration in young men (15). In clinical research, ghrelin and its analogs are typically administered intravenously, either as a bolus or continuous infusion (130), and animal data indicate that such administration allows ghrelin to cross the blood-brain barrier and reach central nervous system targets (38, 128). Other studies reported negative associations, such as poorer memory and language performance in elderly participants with higher ghrelin levels (131), contrasting with animal data. Ghrelin has also been associated with behavioral disinhibition (132), while UAG may counteract the hippocampal effects of AG (see Section 2 for a detailed discussion of AG and UAG). Evidence from patients with Parkinson’s disease and dementia further suggests that the AG: UAG ratio could serve as a circulating biomarker of cognitive decline (133).

Although many studies have examined DS and ghrelin, their interaction with mood and cognition during sleep loss remains insufficiently explored. Notably, mood and cognition appear interconnected: individuals who show stable or improved mood following DS tend to perform better on cognitive assessments compared to those in whom depressive symptoms worsen (5). Given its potential neuroprotective role, ghrelin may represent a mediator of DS-induced mood improvement and associated cognitive changes.

**Figure 1:**
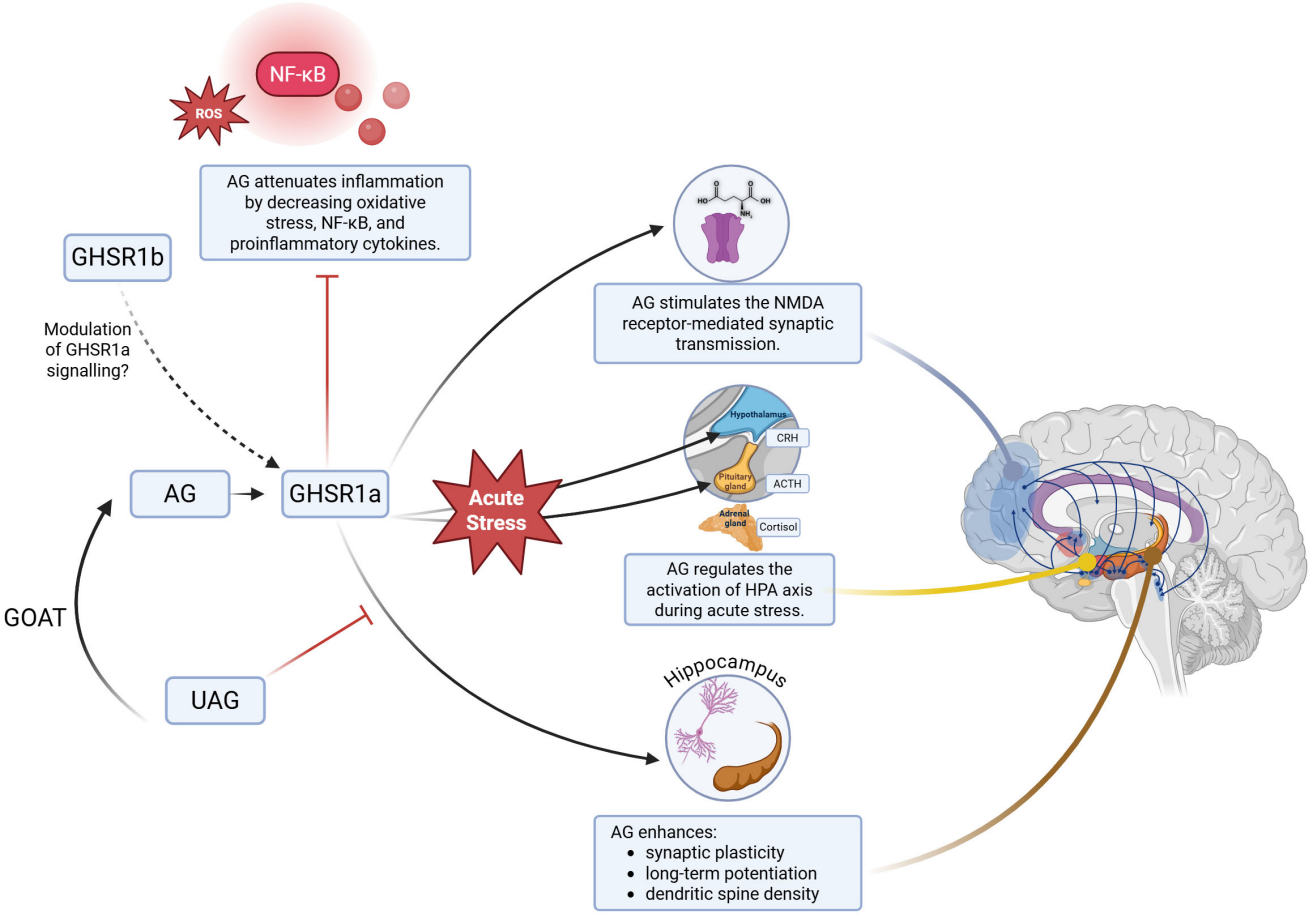
A summary of pathways connecting ghrelin and affective disorders. AG, acyl ghrelin; ACTH, adrenocorticotropic hormone; CRH, corticotropin-releasing hormone; GHSR1a, growth hormone secretagogue receptor type 1a; GHSR1b, growth hormone secretagogue receptor type 1b; GOAT, ghrelin O-acyltransferase; HPA axis, hypothalamus-pituitary-adrenals axis; NMDA, N-methyl-D-aspartate; NK-κB, nuclear factor kappa-light-chain-enhancer of activated B cells; UAG, unacyl ghrelin.

Several pathways are likely to mediate ghrelin’s influence on mood and cognition under DS (**Figure 1**). In response to stress, ghrelin helps regulate the hypothalamic-pituitary-adrenal axis, whose overactivation has been associated with both depression and sleep disturbances (134). Mahbod et al. demonstrated that mice lacking GHSR exhibited significantly higher corticosterone levels than wild-type controls (47), while animals with impaired ghrelin signaling displayed depressive-like behaviors and reduced adaptability to stress (23). These findings suggest that elevated ghrelin levels may serve as a beneficial short-term compensatory mechanism enhancing stress resilience, although its orexigenic effects could increase obesity risk over time (135). Ghrelin may also reduce inflammation by inhibiting the production of pro-inflammatory cytokines (136), an important finding given the strong links between psychiatric disorders and dysregulated inflammatory responses (137). Furthermore, ghrelin affects mood regulation through its actions on the ventral tegmental area (VTA), a region rich in GHSR1a that projects to reward-related structures such as the nucleus accumbens, amygdala, and limbic cortex (22, 138). In addition, ghrelin modulates glutamatergic neurotransmission by regulating NMDA receptor activity. In female mice with depressive-like symptoms, intrahippocampal ghrelin administration reversed NMDA receptor internalization, alleviated depressive behaviors, and improved cognitive performance (139). These findings parallel the rapid antidepressant effects of NMDA antagonists such as ketamine, underscoring the importance of ghrelin signaling in affective disorders.

Overall, ghrelin appears to be a plausible modulator of DS-associated antidepressant and pro-cognitive effects. Proposed mechanisms include promoting adult neurogenesis, enhancing stress resilience (including HPA-axis modulation), dampening neuroinflammation, regulating glutamatergic transmission and synaptic plasticity. Because depressive disorders are often accompanied by cognitive impairments, this dual mood-cognition relevance is particularly salient under circadian misalignment and DS. Ghrelin pathway modulators warrant evaluation as adjuncts to standard care for patients with mood disorders. In parallel, validating biomarker strategies such as the AG: UAG ratio for trial stratification and response monitoring (rather than diagnosis at this stage) could accelerate translation toward biomarker-informed, ghrelin-based interventions for depression.

## 5 Conclusion and future prospects

Ghrelin is a pleiotropic hormone at the intersection of sleep, metabolism, and mental health. It contributes to the regulation of sleep architecture: in human studies, exogenous ghrelin administration has been shown to promote NREM sleep and suppress REM sleep in men, with minimal effects observed in women. This sex-specific difference likely reflects variations in receptor sensitivity rather than differences in circulating exposure alone.

Ghrelin may also play a role in the pathophysiology of sleep disorders. Nocturnal ghrelin levels are typically reduced in insomnia, elevated in obstructive sleep apnea (where they often normalize with CPAP therapy, though findings may be confounded by BMI and hypoxia), and largely unchanged in narcolepsy. These patterns suggest that ghrelin levels are influenced by disorder-specific mechanisms rather than being solely dependent on global alterations in sleep duration or quality.

DS may further alter ghrelin signaling, potentially contributing to the adverse metabolic and cognitive consequences of chronic sleep loss. At the same time, ghrelin could be implicated in the paradoxical antidepressant effect sometimes observed following acute DS. However, evidence remains inconsistent, partly due to methodological heterogeneity, small sample sizes, insufficient to compare ghrelin dynamics between sexes, and lack of differentiation between AG and UAG- an important limitation in many studies assessing circulating ghrelin. Notably, the AG: UAG ratio has emerged as a potential biomarker of dementia risk in patients with Parkinson’s disease, underscoring the importance of distinguishing between these forms, particularly in studies of cognitive impairment.

To summarize, clarifying ghrelin’s contributions to sleep architecture, sleep disorders, and mood regulation may provide valuable insights into its therapeutic potential. While its role in DS-associated mood changes remains preliminary, further research could determine whether targeting ghrelin offers a realistic pathway toward novel, rapid-acting interventions for mood and sleep disorders.
